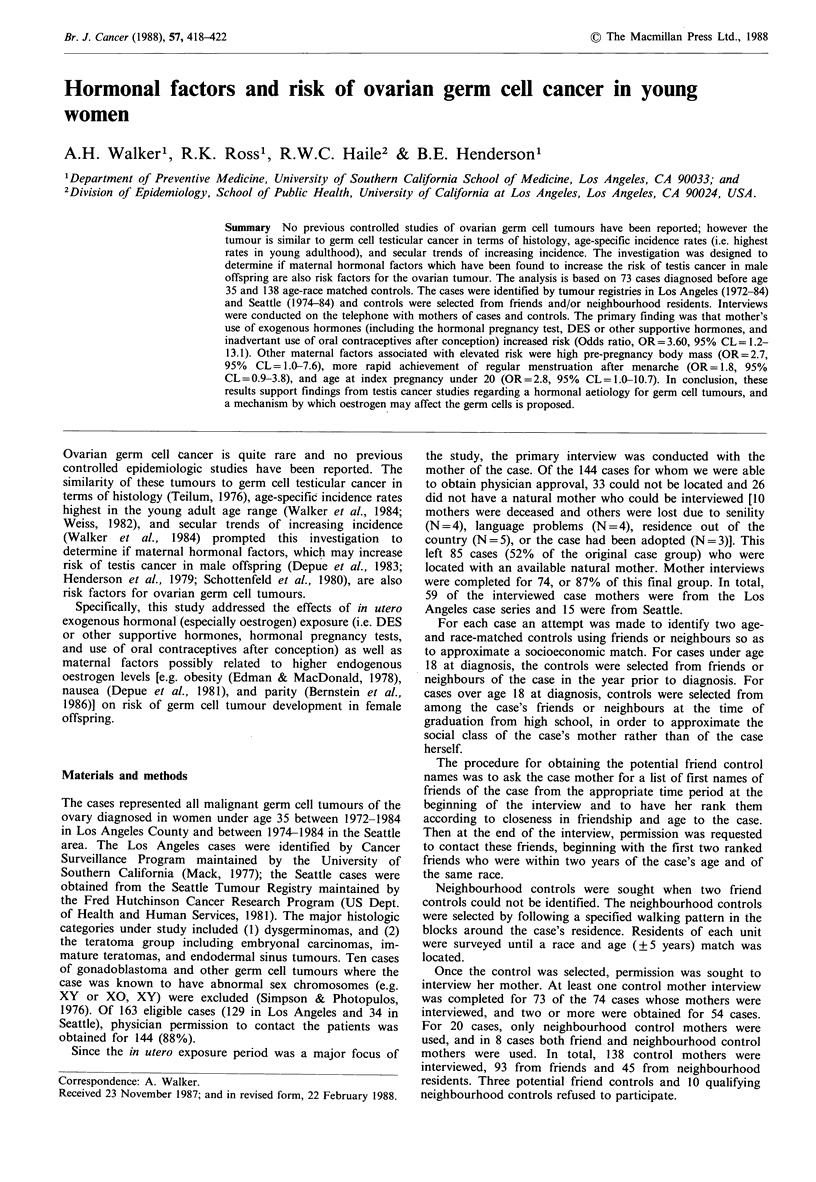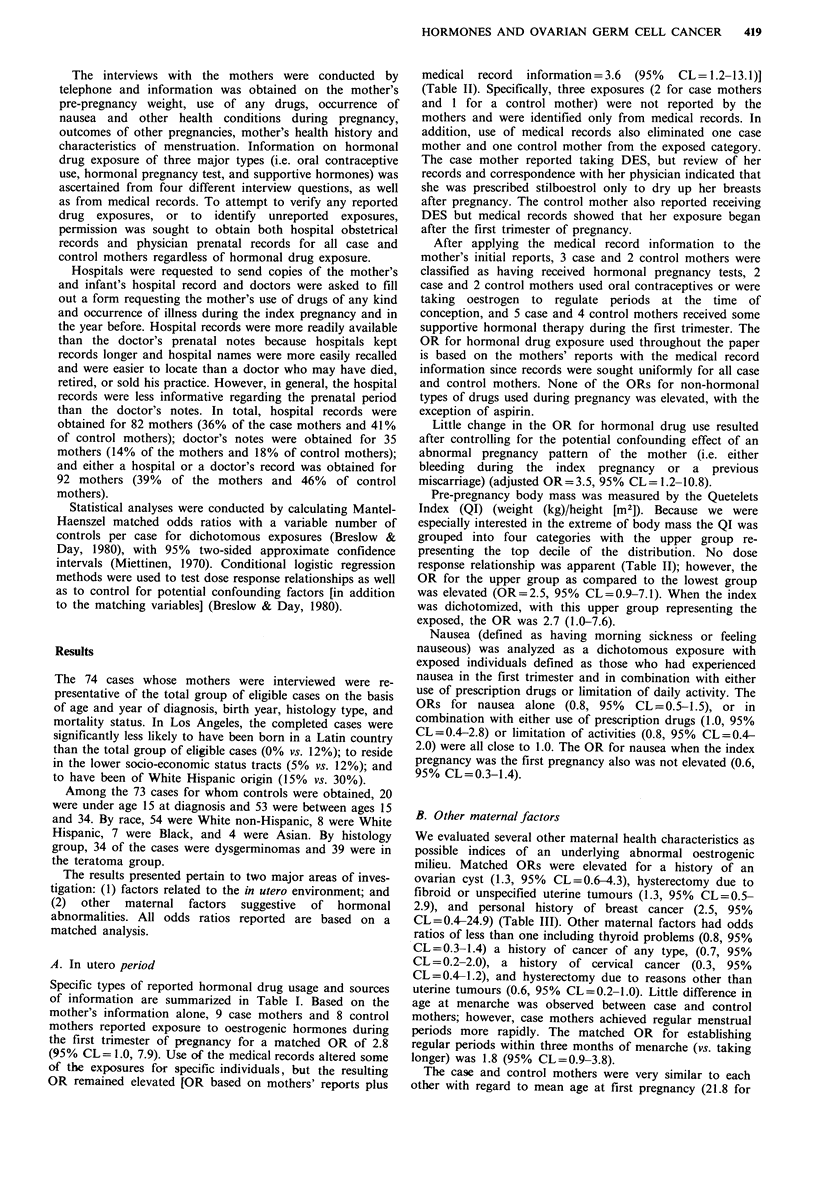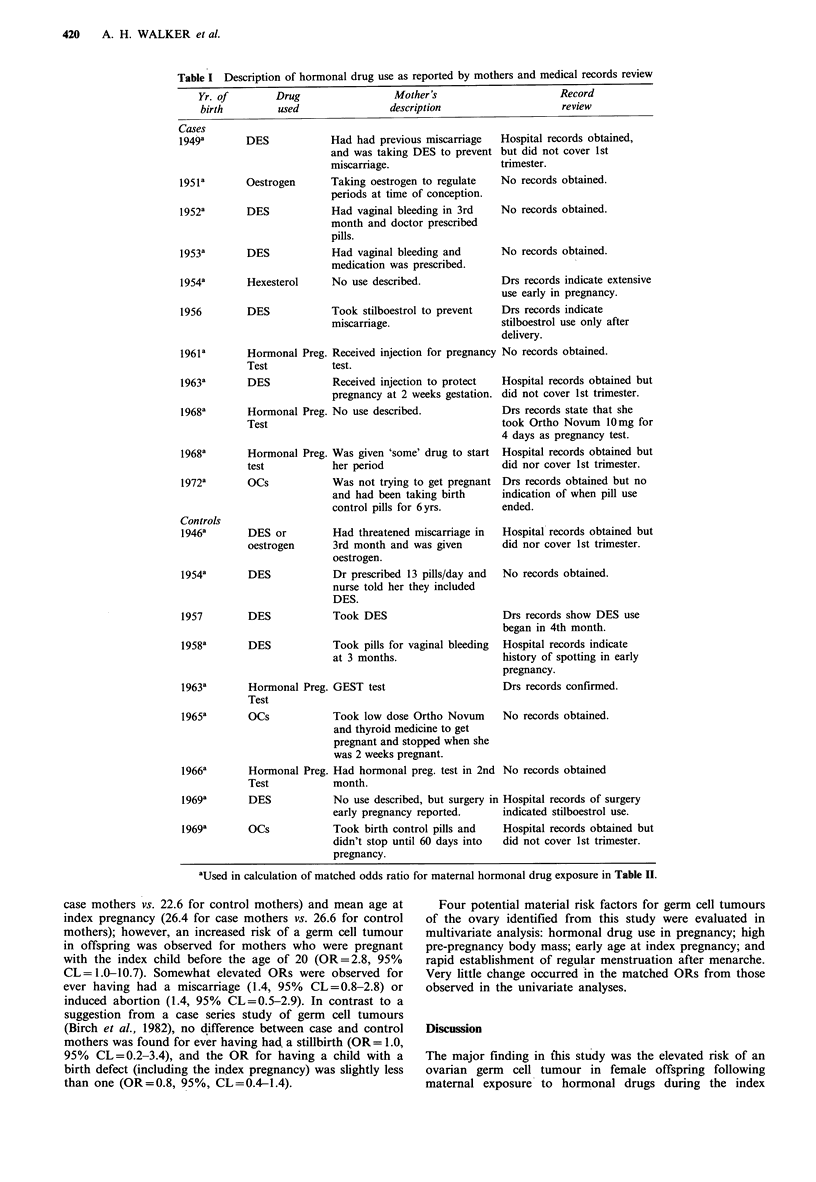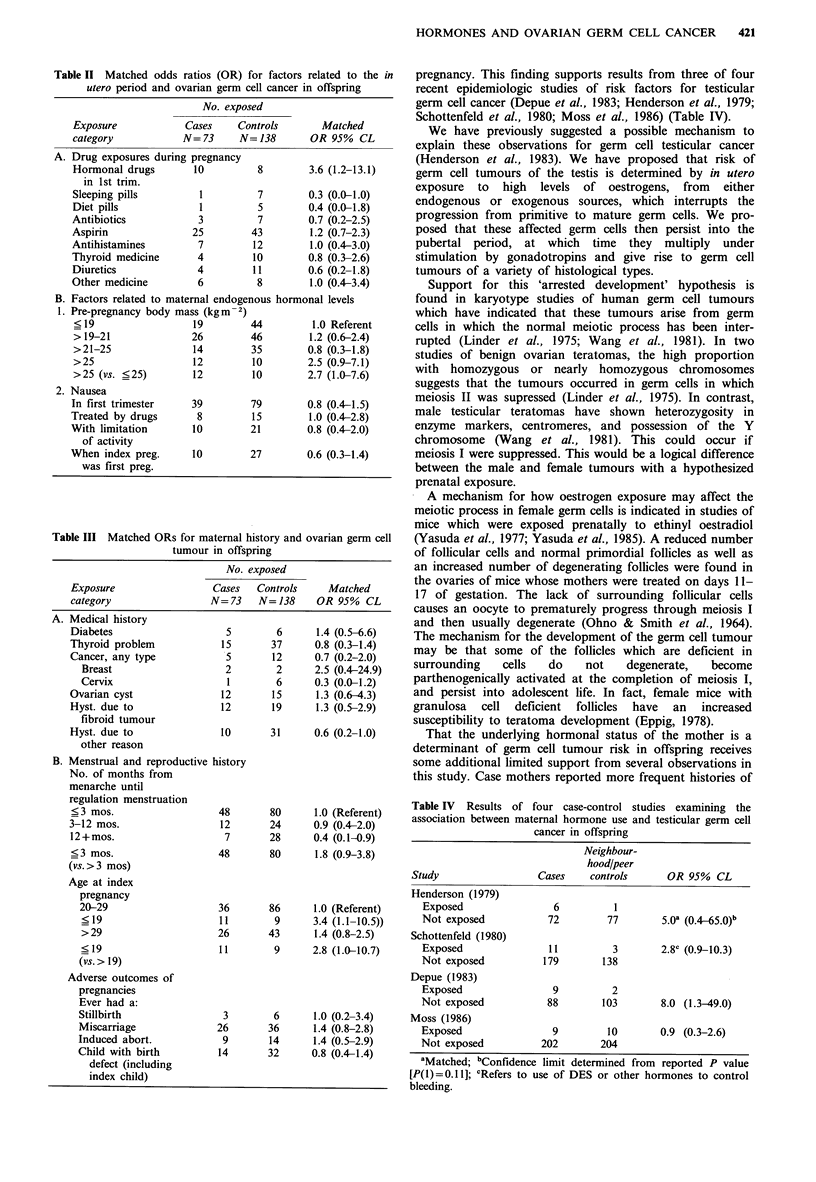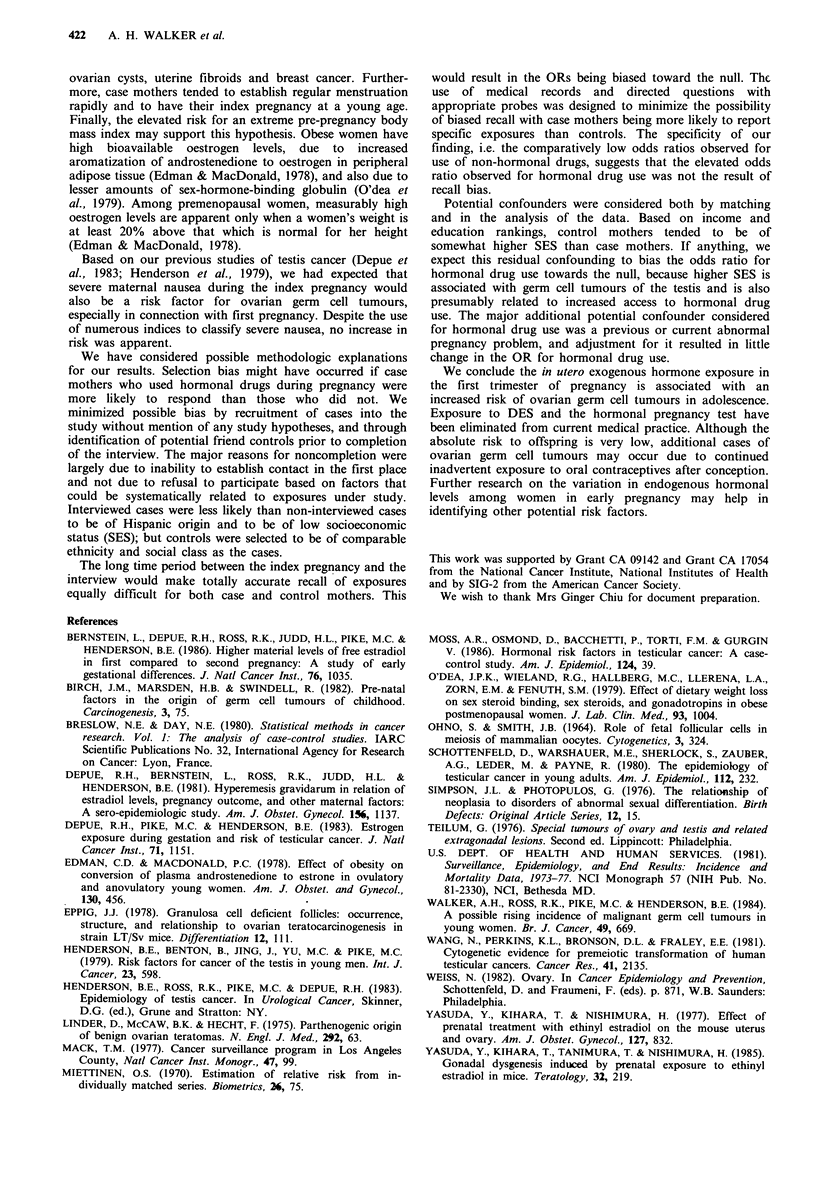# Hormonal factors and risk of ovarian germ cell cancer in young women.

**DOI:** 10.1038/bjc.1988.95

**Published:** 1988-04

**Authors:** A. H. Walker, R. K. Ross, R. W. Haile, B. E. Henderson

**Affiliations:** Department of Preventive Medicine, University of Southern California School of Medicine, Los Angeles 90033.

## Abstract

No previous controlled studies of ovarian germ cell tumours have been reported; however the tumour is similar to germ cell testicular cancer in terms of histology, age-specific incidence rates (i.e. highest rates in young adulthood), and secular trends of increasing incidence. The investigation was designed to determine if maternal hormonal factors which have been found to increase the risk of testis cancer in male offspring are also risk factors for the ovarian tumour. The analysis is based on 73 cases diagnosed before age 35 and 138 age-race matched controls. The cases were identified by tumour registries in Los Angeles (1972-84) and Seattle (1974-84) and controls were selected from friends and/or neighbourhood residents. Interviews were conducted on the telephone with mothers of cases and controls. The primary finding was that mother's use of exogenous hormones (including the hormonal pregnancy test, DES or other supportive hormones, and inadvertant use of oral contraceptives after conception) increased risk (Odds ratio, OR = 3.60, 95% CL = 1.2-13.1). Other maternal factors associated with elevated risk were high pre-pregnancy body mass (OR = 2.7, 95% CL = 1.0-7.6), more rapid achievement of regular menstruation after menarche (OR = 1.8, 95% CL = 0.9-3.8), and age at index pregnancy under 20 (OR = 2.8, 95% CL = 1.0-10.7). In conclusion, these results support findings from testis cancer studies regarding a hormonal aetiology for germ cell tumours, and a mechanism by which oestrogen may affect the germ cells is proposed.


Br. J. Cancer (1988), 57, 418–422 © The Macmillan Press Ltd., 1988

### Hormonal factors and risk of ovarian germ cell cancer in young women

A.H. Walker[1], R.K. Ross[1], R.W.C. Haile[2] & B.E. Henderson[1]

[1]*Department of Preventive Medicine, University of Southern California School of Medicine, Los Angeles, CA 90033; and* [2]*Division of Epidemiology, School of Public Health, University of California at Los Angeles, Los Angeles, CA 90024, USA.*

**Summary** No previous controlled studies of ovarian germ cell tumours have been reported; however the tumour is similar to germ cell testicular cancer in terms of histology, age-specific incidence rates (i.e. highest rates in young adulthood), and secular trends of increasing incidence. The investigation was designed to determine if maternal hormonal factors which have been found to increase the risk of testis cancer in male offspring are also risk factors for the ovarian tumour. The analysis is based on 73 cases diagnosed before age 35 and 138 age-race matched controls. The cases were identified by tumour registries in Los Angeles (1972–84) and Seattle (1974–84) and controls were selected from friends and/or neighbourhood residents. Interviews were conducted on the telephone with mothers of cases and controls. The primary finding was that mother's use of exogenous hormones (including the hormonal pregnancy test, DES or other supportive hormones, and inadvertant use of oral contraceptives after conception) increased risk (Odds ratio, OR = 3.60, 95% CL = 1.2–13.1). Other maternal factors associated with elevated risk were high pre-pregnancy body mass (OR = 2.7, 95% CL = 1.0–7.6), more rapid achievement of regular menstruation after menarche (OR = 1.8, 95% CL = 0.9–3.8), and age at index pregnancy under 20 (OR = 2.8, 95% CL = 1.0–10.7). In conclusion, these results support findings from testis cancer studies regarding a hormonal aetiology for germ cell tumours, and a mechanism by which oestrogen may affect the germ cells is proposed.

Ovarian germ cell cancer is quite rare and no previous controlled epidemiologic studies have been reported. The similarity of these tumours to germ cell testicular cancer in terms of histology (Teilum, 1976), age-specific incidence rates highest in the young adult age range (Walker *et al.*, 1984; Weiss, 1982), and secular trends of increasing incidence (Walker *et al.*, 1984) prompted this investigation to determine if maternal hormonal factors, which may increase risk of testis cancer in male offspring (Depue *et al.*, 1983; Henderson *et al.*, 1979; Schottenfeld *et al.*, 1980), are also risk factors for ovarian germ cell tumours.

Specifically, this study addressed the effects of *in utero* exogenous hormonal (especially oestrogen) exposure (i.e. DES or other supportive hormones, hormonal pregnancy tests, and use of oral contraceptives after conception) as well as maternal factors possibly related to higher endogenous oestrogen levels [e.g. obesity (Edman & MacDonald, 1978), nausea (Depue *et al.*, 1981), and parity (Bernstein *et al.*, 1986)] on risk of germ cell tumour development in female offspring.

### Materials and methods

The cases represented all malignant germ cell tumours of the ovary diagnosed in women under age 35 between 1972–1984 in Los Angeles County and between 1974–1984 in the Seattle area. The Los Angeles cases were identified by Cancer Surveillance Program maintained by the University of Southern California (Mack, 1977); the Seattle cases were obtained from the Seattle Tumour Registry maintained by the Fred Hutchinson Cancer Research Program (US Dept. of Health and Human Services, 1981). The major histologic categories under study included (1) dysgerminomas, and (2) the teratoma group including embryonal carcinomas, immature teratomas, and endodermal sinus tumours. Ten cases of gonadoblastoma and other germ cell tumours where the case was known to have abnormal sex chromosomes (e.g. XY or XO, XY) were excluded (Simpson & Photopulos, 1976). Of 163 eligible cases (129 in Los Angeles and 34 in Seattle), physician permission to contact the patients was obtained for 144 (88%).

Since the *in utero* exposure period was a major focus of the study, the primary interview was conducted with the mother of the case. Of the 144 cases for whom we were able to obtain physician approval, 33 could not be located and 26 did not have a natural mother who could be interviewed [10 mothers were deceased and others were lost due to senility (N = 4), language problems (N = 4), residence out of the country (N = 5), or the case had been adopted (N = 3)]. This left 85 cases (52% of the original case group) who were located with an available natural mother. Mother interviews were completed for 74, or 87% of this final group. In total, 59 of the interviewed case mothers were from the Los Angeles case series and 15 were from Seattle.

For each case an attempt was made to identify two age- and race-matched controls using friends or neighbours so as to approximate a socioeconomic match. For cases under age 18 at diagnosis, the controls were selected from friends or neighbours of the case in the year prior to diagnosis. For cases over age 18 at diagnosis, controls were selected from among the case's friends or neighbours at the time of graduation from high school, in order to approximate the social class of the case's mother rather than of the case herself.

The procedure for obtaining the potential friend control names was to ask the case mother for a list of first names of friends of the case from the appropriate time period at the beginning of the interview and to have her rank them according to closeness in friendship and age to the case. Then at the end of the interview, permission was requested to contact these friends, beginning with the first two ranked friends who were within two years of the case's age and of the same race.

Neighbourhood controls were sought when two friend controls could not be identified. The neighbourhood controls were selected by following a specified walking pattern in the blocks around the case's residence. Residents of each unit were surveyed until a race and age (±5 years) match was located.

Once the control was selected, permission was sought to interview her mother. At least one control mother interview was completed for 73 of the 74 cases whose mothers were interviewed, and two or more were obtained for 54 cases. For 20 cases, only neighbourhood control mothers were used, and in 8 cases both friend and neighbourhood control mothers were used. In total, 138 control mothers were interviewed, 93 from friends and 45 from neighbourhood residents. Three potential friend controls and 10 qualifying neighbourhood controls refused to participate.

Correspondence: A. Walker.
Received 23 November 1987; and in revised form, 22 February 1988.

The interviews with the mothers were conducted by telephone and information was obtained on the mother's pre-pregnancy weight, use of any drugs, occurrence of nausea and other health conditions during pregnancy, outcomes of other pregnancies, mother's health history and characteristics of menstruation. Information on hormonal drug exposure of three major types (i.e. oral contraceptive use, hormonal pregnancy test, and supportive hormones) was ascertained from four different interview questions, as well as from medical records. To attempt to verify any reported drug exposures, or to identify unreported exposures, permission was sought to obtain both hospital obstetrical records and physician prenatal records for all case and control mothers regardless of hormonal drug exposure.

Hospitals were requested to send copies of the mother's and infant's hospital record and doctors were asked to fill out a form requesting the mother's use of drugs of any kind and occurrence of illness during the index pregnancy and in the year before. Hospital records were more readily available than the doctor's prenatal notes because hospitals kept records longer and hospital names were more easily recalled and were easier to locate than a doctor who may have died, retired, or sold his practice. However, in general, the hospital records were less informative regarding the prenatal period than the doctor's notes. In total, hospital records were obtained for 82 mothers (36% of the case mothers and 41% of control mothers); doctor's notes were obtained for 35 mothers (14% of the mothers and 18% of control mothers); and either a hospital or a doctor's record was obtained for 92 mothers (39% of the mothers and 46% of control mothers).

Statistical analyses were conducted by calculating Mantel-Haenszel matched odds ratios with a variable number of controls per case for dichotomous exposures (Breslow & Day, 1980), with 95% two-sided approximate confidence intervals (Miettinen, 1970). Conditional logistic regression methods were used to test dose response relationships as well as to control for potential confounding factors [in addition to the matching variables] (Breslow & Day, 1980).

### Results

The 74 cases whose mothers were interviewed were representative of the total group of eligible cases on the basis of age and year of diagnosis, birth year, histology type, and mortality status. In Los Angeles, the completed cases were significantly less likely to have been born in a Latin country than the total group of eligible cases (0% *vs.* 12%); to reside in the lower socio-economic status tracts (5% *vs.* 12%); and to have been of White Hispanic origin (15% *vs.* 30%).

Among the 73 cases for whom controls were obtained, 20 were under age 15 at diagnosis and 53 were between ages 15 and 34. By race, 54 were White non-Hispanic, 8 were White Hispanic, 7 were Black, and 4 were Asian. By histology group, 34 of the cases were dysgerminomas and 39 were in the teratoma group.

The results presented pertain to two major areas of investigation: (1) factors related to the *in utero* environment; and (2) other maternal factors suggestive of hormonal abnormalities. All odds ratios reported are based on a matched analysis.

### *A.* In utero *period*

Specific types of reported hormonal drug usage and sources of information are summarized in Table I. Based on the mother's information alone, 9 case mothers and 8 control mothers reported exposure to oestrogenic hormones during the first trimester of pregnancy for a matched OR of 2.8 (95% CL = 1.0, 7.9). Use of the medical records altered some of the exposures for specific individuals, but the resulting OR remained elevated [OR based on mothers' reports plus medical record information = 3.6 (95% CL = 1.2–13.1)] (Table II). Specifically, three exposures (2 for case mothers and 1 for a control mother) were not reported by the mothers and were identified only from medical records. In addition, use of medical records also eliminated one case mother and one control mother from the exposed category. The case mother reported taking DES, but review of her records and correspondence with her physician indicated that she was prescribed stilboestrol only to dry up her breasts after pregnancy. The control mother also reported receiving DES but medical records showed that her exposure began after the first trimester of pregnancy.

After applying the medical record information to the mother's initial reports, 3 case and 2 control mothers were classified as having received hormonal pregnancy tests, 2 case and 2 control mothers used oral contraceptives or were taking oestrogen to regulate periods at the time of conception, and 5 case and 4 control mothers received some supportive hormonal therapy during the first trimester. The OR for hormonal drug exposure used throughout the paper is based on the mothers' reports with the medical record information since records were sought uniformly for all case and control mothers. None of the ORs for non-hormonal types of drugs used during pregnancy was elevated, with the exception of aspirin.

Little change in the OR for hormonal drug use resulted after controlling for the potential confounding effect of an abnormal pregnancy pattern of the mother (i.e. either bleeding during the index pregnancy or a previous miscarriage) (adjusted OR = 3.5, 95% CL = 1.2–10.8).

Pre-pregnancy body mass was measured by the Quetelets Index (QI) (weight (kg)/height [$m^2$]). Because we were especially interested in the extreme of body mass the QI was grouped into four categories with the upper group representing the top decile of the distribution. No dose response relationship was apparent (Table II); however, the OR for the upper group as compared to the lowest group was elevated (OR = 2.5, 95% CL = 0.9–7.1). When the index was dichotomized, with this upper group representing the exposed, the OR was 2.7 (1.0–7.6).

Nausea (defined as having morning sickness or feeling nauseous) was analyzed as a dichotomous exposure with exposed individuals defined as those who had experienced nausea in the first trimester and in combination with either use of prescription drugs or limitation of daily activity. The ORs for nausea alone (0.8, 95% CL = 0.5–1.5), or in combination with either use of prescription drugs (1.0, 95% CL = 0.4–2.8) or limitation of activities (0.8, 95% CL = 0.4–2.0) were all close to 1.0. The OR for nausea when the index pregnancy was the first pregnancy also was not elevated (0.6, 95% CL = 0.3–1.4).

### *B. Other maternal factors*

We evaluated several other maternal health characteristics as possible indices of an underlying abnormal oestrogenic milieu. Matched ORs were elevated for a history of an ovarian cyst (1.3, 95% CL = 0.6–4.3), hysterectomy due to fibroid or unspecified uterine tumours (1.3, 95% CL = 0.5–2.9), and personal history of breast cancer (2.5, 95% CL = 0.4–24.9) (Table III). Other maternal factors had odds ratios of less than one including thyroid problems (0.8, 95% CL = 0.3–1.4) a history of cancer of any type, (0.7, 95% CL = 0.2–2.0), a history of cervical cancer (0.3, 95% CL = 0.4–1.2), and hysterectomy due to reasons other than uterine tumours (0.6, 95% CL = 0.2–1.0). Little difference in age at menarche was observed between case and control mothers; however, case mothers achieved regular menstrual periods more rapidly. The matched OR for establishing regular periods within three months of menarche (*vs.* taking longer) was 1.8 (95% CL = 0.9–3.8).

The case and control mothers were very similar to each other with regard to mean age at first pregnancy (21.8 for

**Table I** Description of hormonal drug use as reported by mothers and medical records review

| *Yr. of birth* | *Drug used* | *Mother's description* | *Record review* |
|---|---|---|---|
| *Cases* | | | |
| 1949[a] | DES | Had had previous miscarriage and was taking DES to prevent miscarriage. | Hospital records obtained, but did not cover 1st trimester. |
| 1951[a] | Oestrogen | Taking oestrogen to regulate periods at time of conception. | No records obtained. |
| 1952[a] | DES | Had vaginal bleeding in 3rd month and doctor prescribed pills. | No records obtained. |
| 1953[a] | DES | Had vaginal bleeding and medication was prescribed. | No records obtained. |
| 1954[a] | Hexesterol | No use described. | Drs records indicate extensive use early in pregnancy. |
| 1956 | DES | Took stilboestrol to prevent miscarriage. | Drs records indicate stilboestrol use only after delivery. |
| 1961[a] | Hormonal Preg. Test | Received injection for pregnancy test. | No records obtained. |
| 1963[a] | DES | Received injection to protect pregnancy at 2 weeks gestation. | Hospital records obtained but did not cover 1st trimester. |
| 1968[a] | Hormonal Preg. Test | No use described. | Drs records state that she took Ortho Novum 10 mg for 4 days as pregnancy test. |
| 1968[a] | Hormonal Preg. test | Was given 'some' drug to start her period | Hospital records obtained but did nor cover 1st trimester. |
| 1972[a] | OCs | Was not trying to get pregnant and had been taking birth control pills for 6 yrs. | Drs records obtained but no indication of when pill use ended. |
| *Controls* | | | |
| 1946[a] | DES or oestrogen | Had threatened miscarriage in 3rd month and was given oestrogen. | Hospital records obtained but did nor cover 1st trimester. |
| 1954[a] | DES | Dr prescribed 13 pills/day and nurse told her they included DES. | No records obtained. |
| 1957 | DES | Took DES | Drs records show DES use began in 4th month. |
| 1958[a] | DES | Took pills for vaginal bleeding at 3 months. | Hospital records indicate history of spotting in early pregnancy. |
| 1963[a] | Hormonal Preg. Test | GEST test | Drs records confirmed. |
| 1965[a] | OCs | Took low dose Ortho Novum and thyroid medicine to get pregnant and stopped when she was 2 weeks pregnant. | No records obtained. |
| 1966[a] | Hormonal Preg. Test | Had hormonal preg. test in 2nd month. | No records obtained |
| 1969[a] | DES | No use described, but surgery in early pregnancy reported. | Hospital records of surgery indicated stilboestrol use. |
| 1969[a] | OCs | Took birth control pills and didn't stop until 60 days into pregnancy. | Hospital records obtained but did not cover 1st trimester. |

[a]Used in calculation of matched odds ratio for maternal hormonal drug exposure in **Table II**.

case mothers *vs.* 22.6 for control mothers) and mean age at index pregnancy (26.4 for case mothers *vs.* 26.6 for control mothers); however, an increased risk of a germ cell tumour in offspring was observed for mothers who were pregnant with the index child before the age of 20 (OR=2.8, 95% CL=1.0–10.7). Somewhat elevated ORs were observed for ever having had a miscarriage (1.4, 95% CL=0.8–2.8) or induced abortion (1.4, 95% CL=0.5–2.9). In contrast to a suggestion from a case series study of germ cell tumours (Birch *et al.*, 1982), no difference between case and control mothers was found for ever having had a stillbirth (OR=1.0, 95% CL=0.2–3.4), and the OR for having a child with a birth defect (including the index pregnancy) was slightly less than one (OR=0.8, 95%, CL=0.4–1.4).

Four potential material risk factors for germ cell tumours of the ovary identified from this study were evaluated in multivariate analysis: hormonal drug use in pregnancy; high pre-pregnancy body mass; early age at index pregnancy; and rapid establishment of regular menstruation after menarche. Very little change occurred in the matched ORs from those observed in the univariate analyses.

### Discussion

The major finding in this study was the elevated risk of an ovarian germ cell tumour in female offspring following maternal exposure to hormonal drugs during the index

**Table II** Matched odds ratios (OR) for factors related to the *in utero* period and ovarian germ cell cancer in offspring

| *Exposure category* | *No. exposed* Cases N=73 | Controls N=138 | *Matched OR 95% CL* |
|---|---|---|---|
| A. Drug exposures during pregnancy | | | |
| Hormonal drugs in 1st trim. | 10 | 8 | 3.6 (1.2–13.1) |
| Sleeping pills | 1 | 7 | 0.3 (0.0–1.0) |
| Diet pills | 1 | 5 | 0.4 (0.0–1.8) |
| Antibiotics | 3 | 7 | 0.7 (0.2–2.5) |
| Aspirin | 25 | 43 | 1.2 (0.7–2.3) |
| Antihistamines | 7 | 12 | 1.0 (0.4–3.0) |
| Thyroid medicine | 4 | 10 | 0.8 (0.3–2.6) |
| Diuretics | 4 | 11 | 0.6 (0.2–1.8) |
| Other medicine | 6 | 8 | 1.0 (0.4–3.4) |
| B. Factors related to maternal endogenous hormonal levels | | | |
| 1. Pre-pregnancy body mass (kg m$^{-2}$) | | | |
| ≦19 | 19 | 44 | 1.0 Referent |
| >19–21 | 26 | 46 | 1.2 (0.6–2.4) |
| >21–25 | 14 | 35 | 0.8 (0.3–1.8) |
| >25 | 12 | 10 | 2.5 (0.9–7.1) |
| >25 (*vs.* ≦25) | 12 | 10 | 2.7 (1.0–7.6) |
| 2. Nausea | | | |
| In first trimester | 39 | 79 | 0.8 (0.4–1.5) |
| Treated by drugs | 8 | 15 | 1.0 (0.4–2.8) |
| With limitation of activity | 10 | 21 | 0.8 (0.4–2.0) |
| When index preg. was first preg. | 10 | 27 | 0.6 (0.3–1.4) |

**Table III** Matched ORs for maternal history and ovarian germ cell tumour in offspring

| *Exposure category* | *No. exposed* Cases N=73 | Controls N=138 | *Matched OR 95% CL* |
|---|---|---|---|
| A. Medical history | | | |
| Diabetes | 5 | 6 | 1.4 (0.5–6.6) |
| Thyroid problem | 15 | 37 | 0.8 (0.3–1.4) |
| Cancer, any type | 5 | 12 | 0.7 (0.2–2.0) |
| Breast | 2 | 2 | 2.5 (0.4–24.9) |
| Cervix | 1 | 6 | 0.3 (0.0–1.2) |
| Ovarian cyst | 12 | 15 | 1.3 (0.6–4.3) |
| Hyst. due to fibroid tumour | 12 | 19 | 1.3 (0.5–2.9) |
| Hyst. due to other reason | 10 | 31 | 0.6 (0.2–1.0) |
| B. Menstrual and reproductive history | | | |
| No. of months from menarche until regulation menstruation | | | |
| ≦3 mos. | 48 | 80 | 1.0 (Referent) |
| 3–12 mos. | 12 | 24 | 0.9 (0.4–2.0) |
| 12+mos. | 7 | 28 | 0.4 (0.1–0.9) |
| ≦3 mos. (*vs.*>3 mos) | 48 | 80 | 1.8 (0.9–3.8) |
| Age at index pregnancy | | | |
| 20–29 | 36 | 86 | 1.0 (Referent) |
| ≦19 | 11 | 9 | 3.4 (1.1–10.5)) |
| >29 | 26 | 43 | 1.4 (0.8–2.5) |
| ≦19 (*vs.*>19) | 11 | 9 | 2.8 (1.0–10.7) |
| Adverse outcomes of pregnancies | | | |
| Ever had a: | | | |
| Stillbirth | 3 | 6 | 1.0 (0.2–3.4) |
| Miscarriage | 26 | 36 | 1.4 (0.8–2.8) |
| Induced abort. | 9 | 14 | 1.4 (0.5–2.9) |
| Child with birth defect (including index child) | 14 | 32 | 0.8 (0.4–1.4) |

pregnancy. This finding supports results from three of four recent epidemiologic studies of risk factors for testicular germ cell cancer (Depue *et al.*, 1983; Henderson *et al.*, 1979; Schottenfeld *et al.*, 1980; Moss *et al.*, 1986) (Table IV).

We have previously suggested a possible mechanism to explain these observations for germ cell testicular cancer (Henderson *et al.*, 1983). We have proposed that risk of germ cell tumours of the testis is determined by *in utero* exposure to high levels of oestrogens, from either endogenous or exogenous sources, which interrupts the progression from primitive to mature germ cells. We proposed that these affected germ cells then persist into the pubertal period, at which time they multiply under stimulation by gonadotropins and give rise to germ cell tumours of a variety of histological types.

Support for this 'arrested development' hypothesis is found in karyotype studies of human germ cell tumours which have indicated that these tumours arise from germ cells in which the normal meiotic process has been interrupted (Linder *et al.*, 1975; Wang *et al.*, 1981). In two studies of benign ovarian teratomas, the high proportion with homozygous or nearly homozygous chromosomes suggests that the tumours occurred in germ cells in which meiosis II was supressed (Linder *et al.*, 1975). In contrast, male testicular teratomas have shown heterozygosity in enzyme markers, centromeres, and possession of the Y chromosome (Wang *et al.*, 1981). This could occur if meiosis I were suppressed. This would be a logical difference between the male and female tumours with a hypothesized prenatal exposure.

A mechanism for how oestrogen exposure may affect the meiotic process in female germ cells is indicated in studies of mice which were exposed prenatally to ethinyl oestradiol (Yasuda *et al.*, 1977; Yasuda *et al.*, 1985). A reduced number of follicular cells and normal primordial follicles as well as an increased number of degenerating follicles were found in the ovaries of mice whose mothers were treated on days 11–17 of gestation. The lack of surrounding follicular cells causes an oocyte to prematurely progress through meiosis I and then usually degenerate (Ohno & Smith *et al.*, 1964). The mechanism for the development of the germ cell tumour may be that some of the follicles which are deficient in surrounding cells do not degenerate, become parthenogenically activated at the completion of meiosis I, and persist into adolescent life. In fact, female mice with granulosa cell deficient follicles have an increased susceptibility to teratoma development (Eppig, 1978).

That the underlying hormonal status of the mother is a determinant of germ cell tumour risk in offspring receives some additional limited support from several observations in this study. Case mothers reported more frequent histories of

**Table IV** Results of four case-control studies examining the association between maternal hormone use and testicular germ cell cancer in offspring

| *Study* | *Cases* | *Neighbourhood/peer controls* | *OR 95% CL* |
|---|---|---|---|
| Henderson (1979) | | | |
| Exposed | 6 | 1 | |
| Not exposed | 72 | 77 | 5.0[a] (0.4–65.0)[b] |
| Schottenfeld (1980) | | | |
| Exposed | 11 | 3 | 2.8[c] (0.9–10.3) |
| Not exposed | 179 | 138 | |
| Depue (1983) | | | |
| Exposed | 9 | 2 | |
| Not exposed | 88 | 103 | 8.0 (1.3–49.0) |
| Moss (1986) | | | |
| Exposed | 9 | 10 | 0.9 (0.3–2.6) |
| Not exposed | 202 | 204 | |

[a]Matched; [b]Confidence limit determined from reported *P* value [*P*(1)=0.11]; [c]Refers to use of DES or other hormones to control bleeding.

ovarian cysts, uterine fibroids and breast cancer. Furthermore, case mothers tended to establish regular menstruation rapidly and to have their index pregnancy at a young age. Finally, the elevated risk for an extreme pre-pregnancy body mass index may support this hypothesis. Obese women have high bioavailable oestrogen levels, due to increased aromatization of androstenedione to oestrogen in peripheral adipose tissue (Edman & MacDonald, 1978), and also due to lesser amounts of sex-hormone-binding globulin (O'dea *et al.*, 1979). Among premenopausal women, measurably high oestrogen levels are apparent only when a women's weight is at least 20% above that which is normal for her height (Edman & MacDonald, 1978).

Based on our previous studies of testis cancer (Depue *et al.*, 1983; Henderson *et al.*, 1979), we had expected that severe maternal nausea during the index pregnancy would also be a risk factor for ovarian germ cell tumours, especially in connection with first pregnancy. Despite the use of numerous indices to classify severe nausea, no increase in risk was apparent.

We have considered possible methodologic explanations for our results. Selection bias might have occurred if case mothers who used hormonal drugs during pregnancy were more likely to respond than those who did not. We minimized possible bias by recruitment of cases into the study without mention of any study hypotheses, and through identification of potential friend controls prior to completion of the interview. The major reasons for noncompletion were largely due to inability to establish contact in the first place and not due to refusal to participate based on factors that could be systematically related to exposures under study. Interviewed cases were less likely than non-interviewed cases to be of Hispanic origin and to be of low socioeconomic status (SES); but controls were selected to be of comparable ethnicity and social class as the cases.

The long time period between the index pregnancy and the interview would make totally accurate recall of exposures equally difficult for both case and control mothers. This would result in the ORs being biased toward the null. The use of medical records and directed questions with appropriate probes was designed to minimize the possibility of biased recall with case mothers being more likely to report specific exposures than controls. The specificity of our finding, i.e. the comparatively low odds ratios observed for use of non-hormonal drugs, suggests that the elevated odds ratio observed for hormonal drug use was not the result of recall bias.

Potential confounders were considered both by matching and in the analysis of the data. Based on income and education rankings, control mothers tended to be of somewhat higher SES than case mothers. If anything, we expect this residual confounding to bias the odds ratio for hormonal drug use towards the null, because higher SES is associated with germ cell tumours of the testis and is also presumably related to increased access to hormonal drug use. The major additional potential confounder considered for hormonal drug use was a previous or current abnormal pregnancy problem, and adjustment for it resulted in little change in the OR for hormonal drug use.

We conclude the *in utero* exogenous hormone exposure in the first trimester of pregnancy is associated with an increased risk of ovarian germ cell tumours in adolescence. Exposure to DES and the hormonal pregnancy test have been eliminated from current medical practice. Although the absolute risk to offspring is very low, additional cases of ovarian germ cell tumours may occur due to continued inadvertent exposure to oral contraceptives after conception. Further research on the variation in endogenous hormonal levels among women in early pregnancy may help in identifying other potential risk factors.

This work was supported by Grant CA 09142 and Grant CA 17054 from the National Cancer Institute, National Institutes of Health and by SIG-2 from the American Cancer Society.

We wish to thank Mrs Ginger Chiu for document preparation.

### References

BERNSTEIN, L., DEPUE, R.H., ROSS, R.K., JUDD, H.L., PIKE, M.C. & HENDERSON, B.E. (1986). Higher material levels of free estradiol in first compared to second pregnancy: A study of early gestational differences. *J. Natl Cancer Inst.*, **76**, 1035.

BIRCH, J.M., MARSDEN, H.B. & SWINDELL, R. (1982). Pre-natal factors in the origin of germ cell tumours of childhood. *Carcinogenesis*, **3**, 75.

BRESLOW, N.E. & DAY, N.E. (1980). *Statistical methods in cancer research. Vol. 1: The analysis of case-control studies.* IARC Scientific Publications No. 32, International Agency for Research on Cancer: Lyon, France.

DEPUE, R.H., BERNSTEIN, L., ROSS, R.K., JUDD, H.L. & HENDERSON, B.E. (1981). Hyperemesis gravidarum in relation of estradiol levels, pregnancy outcome, and other maternal factors: A sero-epidemiologic study. *Am. J. Obstet. Gynecol.* **156**, 1137.

DEPUE, R.H., PIKE, M.C. & HENDERSON, B.E. (1983). Estrogen exposure during gestation and risk of testicular cancer. *J. Natl Cancer Inst.*, **71**, 1151.

EDMAN, C.D. & MACDONALD, P.C. (1978). Effect of obesity on conversion of plasma androstenedione to estrone in ovulatory and anovulatory young women. *Am. J. Obstet. and Gynecol.*, **130**, 456.

EPPIG, J.J. (1978). Granulosa cell deficient follicles: occurrence, structure, and relationship to ovarian teratocarcinogenesis in strain LT/Sv mice. *Differentiation* **12**, 111.

HENDERSON, B.E., BENTON, B., JING, J., YU, M.C. & PIKE, M.C. (1979). Risk factors for cancer of the testis in young men. *Int. J. Cancer*, **23**, 598.

HENDERSON, B.E., ROSS, R.K., PIKE, M.C. & DEPUE, R.H. (1983). Epidemiology of testis cancer. In *Urological Cancer*, Skinner, D.G. (ed.), Grune and Stratton: NY.

LINDER, D., McCAW, B.K. & HECHT, F. (1975). Parthenogenic origin of benign ovarian teratomas. *N. Engl. J. Med.*, **292**, 63.

MACK, T.M. (1977). Cancer surveillance program in Los Angeles County, *Natl Cancer Inst. Monogr.*, **47**, 99.

MIETTINEN, O.S. (1970). Estimation of relative risk from individually matched series. *Biometrics*, **26**, 75.

MOSS, A.R., OSMOND, D., BACCHETTI, P., TORTI, F.M. & GURGIN V. (1986). Hormonal risk factors in testicular cancer: A case-control study. *Am. J. Epidemiol.*, **124**, 39.

O'DEA, J.P.K., WIELAND, R.G., HALLBERG, M.C., LLERENA, L.A., ZORN, E.M. & FENUTH, S.M. (1979). Effect of dietary weight loss on sex steroid binding, sex steroids, and gonadotropins in obese postmenopausal women. *J. Lab. Clin. Med.*, **93**, 1004.

OHNO, S. & SMITH, J.B. (1964). Role of fetal follicular cells in meiosis of mammalian oocytes. *Cytogenetics*, **3**, 324.

SCHOTTENFELD, D., WARSHAUER, M.E., SHERLOCK, S., ZAUBER, A.G., LEDER, M. & PAYNE, R. (1980). The epidemiology of testicular cancer in young adults. *Am. J. Epidemiol.*, **112**, 232.

SIMPSON, J.L. & PHOTOPULOS, G. (1976). The relationship of neoplasia to disorders of abnormal sexual differentiation. *Birth Defects: Original Article Series*, **12**, 15.

TEILUM, G. (1976). *Special tumours of ovary and testis and related extragonadal lesions.* Second ed. Lippincott: Philadelphia.

U.S. DEPT. OF HEALTH AND HUMAN SERVICES. (1981). *Surveillance, Epidemiology, and End Results: Incidence and Mortality Data, 1973–77.* NCI Monograph 57 (NIH Pub. No. 81-2330), NCI, Bethesda MD.

WALKER, A.H., ROSS, R.K., PIKE, M.C. & HENDERSON, B.E. (1984). A possible rising incidence of malignant germ cell tumours in young women. *Br. J. Cancer*, **49**, 669.

WANG, N., PERKINS, K.L., BRONSON, D.L. & FRALEY, E.E. (1981). Cytogenetic evidence for premeiotic transformation of human testicular cancers. *Cancer Res.*, **41**, 2135.

WEISS, N. (1982). Ovary. In *Cancer Epidemiology and Prevention*, Schottenfeld, D. and Fraumeni, F. (eds). p. 871, W.B. Saunders: Philadelphia.

YASUDA, Y., KIHARA, T. & NISHIMURA, H. (1977). Effect of prenatal treatment with ethinyl estradiol on the mouse uterus and ovary. *Am. J. Obstet. Gynecol.*, **127**, 832.

YASUDA, Y., KIHARA, T., TANIMURA, T. & NISHIMURA, H. (1985). Gonadal dysgenesis induced by prenatal exposure to ethinyl estradiol in mice. *Teratology*, **32**, 219.